# Effect of Age and Lordotic Angle on the Level of Lumbar Disc Herniation

**DOI:** 10.4061/2011/950576

**Published:** 2011-09-07

**Authors:** Ghassan S. Skaf, Chakib M. Ayoub, Nathalie T. Domloj, Massud J. Turbay, Cherine El-Zein, Mukbil H. Hourani

**Affiliations:** ^1^Division of Neurosurgery, Department of Surgery, American University of Beirut Medical Center, P.O. Box 11-0236, Riad El Solh, Beirut 1107 2020, Lebanon; ^2^Department of Anesthesiology, American University of Beirut Medical Center, P.O. Box 11-0236, Riad El Solh, Beirut 1107 2020, Lebanon; ^3^Department of Radiology, American University of Beirut Medical Center, P.O. Box 11-0236, Riad El Solh, Beirut 1107 2020, Lebanon

## Abstract

It has been previously suggested in the literature that with aging, degenerative changes as well as disc herniation start at the lower lumbar segments, with higher disc involvement observed in an ascending fashion in older age groups. We conducted a study to investigate this correlation between age and level of disc herniation, and to associate it with the magnitude of the Lumbar Lordotic Angle (LLA), as measured by Cobb's method. We followed retrospectively lumbosacral spine MRI's of 1419 patients with symptomatic disc herniation. Pearson's correlation was used in order to investigate the relationship between LLA, age, and level of disc herniation. Student's *t*-test was applied to assess gender differences. Young patients were found to have higher LLA (*R* = 0.44, *P* < 0.0001) and lower levels of disc herniation (*R* = 0.302, *P* < 0.0001), whereas older patients had higher level herniation in lower LLA group (mean LLA 28.6° and 25.4°) and lower level herniation in high LLA group (mean LLA 33.2°). We concluded that Lumbar lordotic Cobb's angle and age can be predictors of the level of lumbar disc herniation. This did not differ among men and women (*R* = 0.341, *P* < 0.0001).

## 1. Introduction

The mechanical properties of the intervertebral discs, as well as the interplay between the vertebral spine curvature and the ligaments and musculature that maintain it, not only impart a resilience that is important in protecting the vertebral column against compressive forces encountered in various postures [1], but largely determine the changes induced by aging, which are subsequently associated with degeneration [2], tropism [3, 4], disc herniation [5], and lordosis [6]. These variations are level [7] as well as gender specific [8].

Published morphometric studies suggest that lumbar disc herniation is more cranially localized with age [5], and this finding is as well a mirror spread of degenerative changes [9] which start earlier at the lower lumbar levels [10].

The vertebral spine presents regional curves on sagittal plane, designed to absorb impact, reduce its longitudinal stiffness, and intensify muscular function; nevertheless, it has been noted that some cases of low back pain and sciatica are attributable to abnormal alterations of the curve [11].

 Lumbar lordosis is defined as the curvature assumed by the intact lumbar spine to compensate for the inclination of the sacrum, restore an upward spinal orientation, and consequently avoid a forward inclination. Its measure, as proven by multiple investigators, is influenced by various parameters, including age, gender, pelvic bend, and thoracic curvature, among others [7, 8]. Its angle was found to increase with age [12], with no differences between men and women, and has been further correlated to intervertebral stress distribution [13], facet angle and orientation [4, 14], and trunk muscles imbalance [11].

The present study was undertaken to reveal that not only disc herniation occurred at higher levels of the spine with a high age group, and a low lordotic lumbar Cobb's angle, but also to emphasize that if the latter were to be altered; it would implicate a different level of disc herniation as expected by age group.

## 2. Materials and Methods

Lumbosacral spine MRIs of 2247 patients suffering from low back and sciatic pain were collected retrospectively. Clinical data was obtained from the American University of Beirut Medical Center neurological archive between 2002 and 2005. Subjects were healthy men and women, with ages ranging from 19 to 84 years old. 63% of the patients (*N* = 1419) had evidence of lumbar disc herniation, 25% showed disc degeneration without herniation at one or more levels (*N* = 560), and 12% had normal MRIs (*N* = 268). 

Radiographs from the 1419 individuals with disc herniation were examined following Institutional Review Board approval. Records included age, sex, and number and level of disc herniation, number and level of disc degeneration (when more than one herniated disc was found, the level included in the study was the symptomatic one).

**Figure 1:**
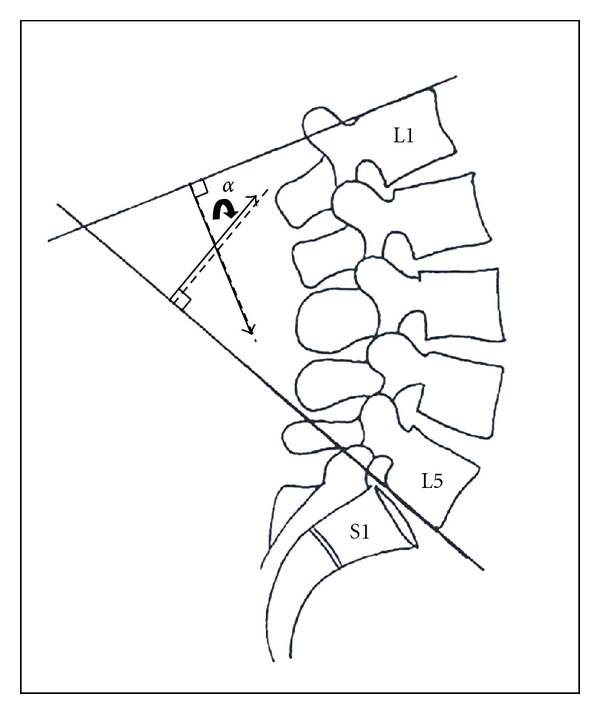
Measurement of lumbar lordotic angle *α*. Using Cobb's method, tangent lines are drawn along the superior end pated of L1 and S1. Perpendiculars to each of the lines are added to form *α*.

Lumbar lordotic angle (LLA) was measured using Cobb's method (Figure 1), by an independent radiologist who had no access to the clinical data. T2-weighted mid-sagittal MRI images were read using as reference L1 and S1 vertebral bodies, where lines were drawn along their superior end plates, to extend past the vertebral body. Perpendiculars were then added, on the side of convergence of the two lines, and the angle of the intersection of these perpendiculars was measured, forming Cobb's angle, thereby giving a global estimate of lumbar lordosis. 

The Pearson's correlation coefficients between lumbar lordosis, age, and level of disc herniation were calculated by the SPSS for Windows 13.0. Correlation was determined using standard linear regression. Student's *t*-test was used to compare the different parameters among men and women. *P* < 0.05 was considered statistically significant.

## 3. Results

### 3.1. Disc Herniation

**Figure 2:**
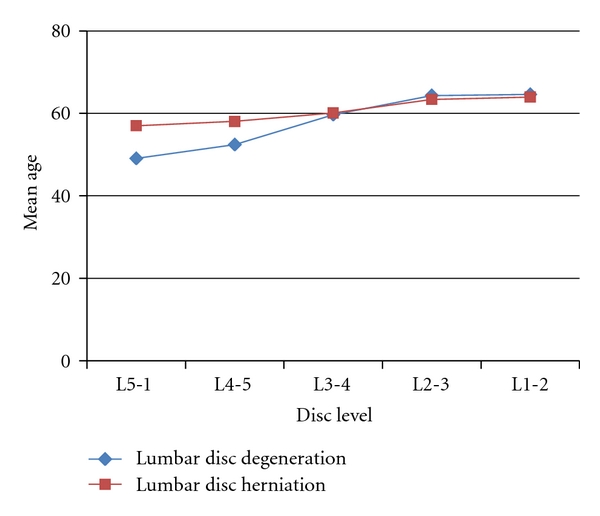
Lumbar disc herniation and lumbar disc degeneration versus age. The numbers in the figure correspond to different mean ages (±SEM) with respect to disc herniation and degeneration levels. The level of lumbar disc herniation and degeneration increased with age.

There were 723 men (mean age = 51.7) and 696 women (mean age = 54.7). The subjects' mean ages (±SEM) with respect to the levels of lumbar herniated discs were measured and plotted (Figure 2). The mean age of patients with disc herniation at levels L3-4, L2-3, and L1-2 was significantly higher than the mean age of patients with disc herniation at lower levels, meaning L4-5, L5-S1 (*P* < 0.0001).

**Figure 3:**
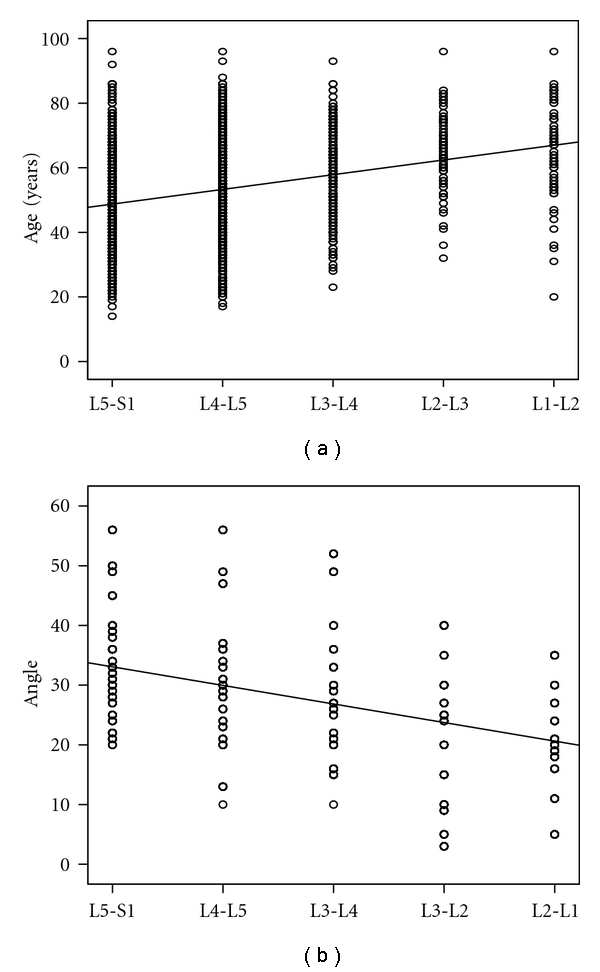
Level of lumbar disc herniation versus age and lumbar lordotic Cobb's angle. (a) The numbers in the figure correspond to different mean ages (±SEM) with respect to disc herniation levels. There is a significant increase in age as the lumbar disc herniation moves in the cranial direction. (b) The numbers in the figure correspond to the measured mean angles (±SEM) at different lumbar levels. The angle tends to decrease according to the lumbar disc herniation level in the cranial direction.

The level of lumbar disc herniation was further found to increase in the cranial direction with age (*R* = 0.302, *P* < 0.0001), as represented by standard linear regression (Figure 3(a)), and this correlation did not differ among men and women when evaluated by Student's *t*-test. 

To further study this association, patients with symptomatic disc herniation (*N* = 1419) were classified according to decades of life. Older age groups assumed larger frequencies of high level lumbar disc herniations (L1-2, L2-3, L3-4), whereas younger age groups (10–19, 20–29, 30–39, 40–49) had a tendency to shift towards lower levels of lumbar disc herniations (L5-S1, L4-5). An alternative interpretation of the graphs revealed that a percentage increase in disc herniation was evident at the lower lumber levels (L1-2, L2-3, L3-4) while patients grew older, with the highest frequency being at the sixth decade of life, as compared to other age groups, who showed relatively constant results with aging (L4-5), or a gradual decrease in disc herniation along with age, to become substantial after the sixth decade (L5-S1).

### 3.2. Lumbar Curvature Angle (Lumbar Lordosis)

**Figure 4:**
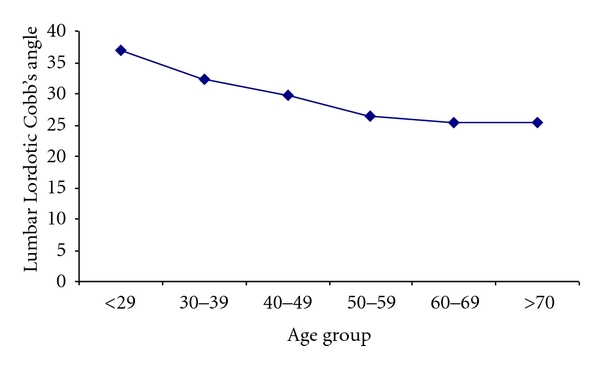
Lumbar lordotic Cobb's angle and decades of life. The numbers in the figure correspond to the mean angles (±SEM) at different decade of life. It shows a decrease in the magnitude of LLA with age.

The Lumbar lordotic angle (LLA) as measured by Cobb's method did not significantly differ among men (28.39° ± 0.47° (SEM)) and women (28.59° ± 0.43° (SEM)) (*R* = 0.01, *P* > 0.05). However, it significantly correlated with age (*R* = 0.341, *P* < 0.0001) and showed a tendency to decrease from the third decade onwards, to become relatively constant after the sixth decade (Figure 4).

Similarly, lumbar lordotic Cobb's angle tended to be level specific and decreased as lumbar disc herniation level moved in the cranial direction, as evidenced by standard linear regression (*R* = 0.44, *P* < 0.0001) (Figure 3(b)). 

In order to assess the influence of the lumbar lordotic angle on the level of disc herniation as it correlated to age, patients with lumbar disc herniation levels outside the expected range according to their relative age groups were evaluated as well. We compared two groups of patients within the 30–39 (*N* = 223), 40–49 (*N* = 308), and >70 (*N* = 218) age groups. The remaining patients within the other age groups were discarded since they represented a minimal number of cases. Group 1 comprised the patients who showed unexpected levels of disc herniation according to their age groups (5, 11, and 115, resp.), and group 2 included those who had normal herniated disc levels as expected by age.  Table 1 compares the different angles measured for each of those individuals.

Patients in group 1 who were young (*N* = 5, *N* = 11) had unexpectedly high levels of lumbar disc herniation (L1-2, L2-3). Their mean lumbar lordotic Cobb's angles were also found to be lower than predicted, as compared to group 2. Similarly, older patients in group 1 (*N* = 115), who showed low levels of lumbar disc herniation (L4-5, L5-S1), had higher lumbar lordotic angle measurements than expected.

### 3.3. Disc Degeneration

The number of degenerated discs among our population sample varied between 0 and 5. The mean ages (±SEM) at the different levels of disc degeneration are shown (Figure 2). It was evident that the same correlation found earlier between age and level of disc herniation applied to disc degeneration, as the mean age gradually increased according to the disc degeneration level in the cranial direction (*P* < 0.0001). 

**Figure 5:**
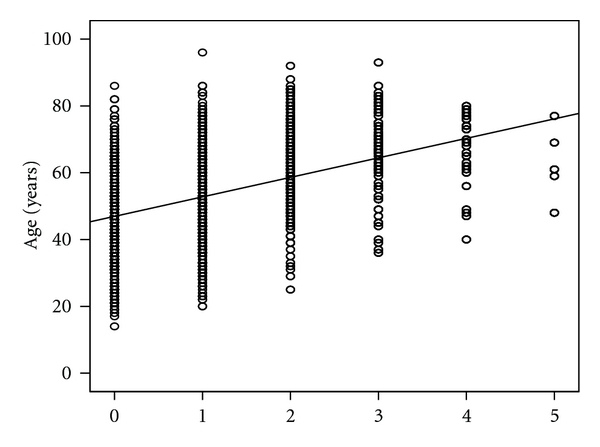
Number of disc degeneration and age. The numbers in the figure correspond to different mean ages (±SEM) with respect to the number of degenerated discs. There is a significant increase in that number with aging.

Furthermore, the number of degenerated discs increased with age as shown by standard linear regression (*R* = 0.302, *P* < 0.0001) (Figure 5). 

In addition, when grouping patients by sex, Student's *t*-test revealed no significant difference among men and women concerning the number and the level of degenerated discs.

## 4. Discussion

Nowadays, it is estimated that at one time, 80% of the population will be afflicted with low back pain [15, 16]. Disc herniation, and degeneration which were found to increase with age [1, 5] were reported to be significant factors in the genesis of it. These changes can be attributed to trunk muscles imbalance [11], hypolordosis [6, 12], intervertebral stress distribution [13], and facet tropism [17, 18]. 

It is commonly accepted that trunk musculature and intra-abdominal pressure produced by muscular activity stabilize spinal structures [11, 19]. The abdominal muscles originate from the crest and symphysis pubis and insert on the xyphoid process and cartilages of five to seven ribs anatomically and therefore can tilt the pelvis posteriorly, and hence they change the curvature of the lumbar spine. Consequently, it has been hypothesized that with aging, an imbalance of trunk muscle due to weakness of abdominal muscles can increase the lordotic curvature of the lumbar spine [11]. As per our findings, the measure of the lumbar lordotic Cobb's angle decreased with age, which is parallel to an increase in lumbar lordosis.

VonLackum in 1924 showed that an increase in lordotic angle proportionally increases the shearing strain or stress in the anterior direction and shifts the center of gravity anteriorly. Both mechanics will increase the shearing strain at the lumbosacral junction [20]. This increased angle and stress is thought by some to be associated with poor posture and back pain, leading subsequently to a decrease in the lumbar lordotic Cobb's angle.

Furthermore, the mechanical stresses acting within intervertebral discs are relevant as well to the interpretation of our findings. The central region of a lumbar intervertebral disc behaves like a pressurized fluid [21], with the highest compressive stresses found in the annulus. Age-related degenerative changes are probably initiated by damage to the annulus or endplate [22], or by a cell-mediated reduction in proteoglycan content. Either of these would reduce the nuclear pressure and transfer compressive stress from nucleus pulposus to posterior annulus, which may be a cause of pain [13, 23]. These disruptive changes were believed to start earlier at the lower lumbar levels [2, 7].

Facet joints have a significant role in providing stability to the spine and controlling motion in six degrees of freedom under the complex loading of the spine [17]. Tropism is defined as asymmetry in the left and right facet joint angles of the lumbar spine. Some authors suggest a strong relation between facet tropism and disc degeneration [4, 24]. Ko and Park noted that the cadaver specimens with tropism tended to rotate toward the more oblique facet when an axial load was placed on the spine segment. The rotation resulting from the joint asymmetry can place additional torsional stress on the annulus fibrosus, contributing to injury [17]. This rotation represents a possible mechanism for the increased incidence of disc degeneration seen in the presence of tropism. It is as well generally accepted that in addition to intervertebral disc degeneration, mechanical stress plays an important role in the development of intervertebral disc herniation. Farfan and Sullivan emphasized that torsional stress of a magnitude encountered in daily activity plays a major role in the initiation of disc degeneration and herniation. They suggested that asymmetry of the facet joints correlated with the development of disc herniation, because the coronary facing facet joint offers little resistance to intervertebral shear force, so the rotation occurs toward the side of the more coronary facing facet joint, and this possibly leads to additional torsional stress on the annulus fibrosus [3].

Similarly in our study, it was suggested that with aging, degenerative changes as well as disc herniation start at the lower lumbar segments. Indeed, it has been demonstrated that changes in the mechanical characteristics of the discs are greater in those that are adjacent to fused lumbar vertebrae, favoring degeneration, with high disc involvement observed more commonly in an ascending fashion in older age groups. Taylor et al. [25] evaluated and confirmed the hypothesis that with aging the loss of proteoglycans from the lumbosacral disc exceeds that from the upper lumbar discs because of its proximity to a rigid segment, that is, the sacrum. This relative fixation and associated facet arthropathy results in greater stresses at the more rostral angles, leading to disc herniation, with it moving cranially, a mirror spread of disc degeneration. 

Furthermore, it is obvious that with aging, the loss of trunk muscles balance, along with an increase in lumbar lordotic angle (decrease in lordosis), would lead to an increase in intervertebral disc stresses with an eventual change in pressure points along the lumbar spine to be moving upwards. Moreover, symmetrical facets distribute a given load evenly and bilaterally. With increasingly oblique angles, the facets in the lumbar spine become less effective in resisting rotational forces. The loading force in asymmetrical facet joints can be shifted to the side of the facet joint, and the subsequent eccentric stress will affect the disc, resulting in disc degeneration or herniation [14]. These factors would implicate the patterns of change in levels of lumbar disc degeneration and herniation to be moving cranially with age. Consequently, any modification in the lordotic lumbar angle would lead to a change in the level of lumbar disc herniation as accounted for by age, as exemplified by our patient group that had unexpected levels of lumbar disc herniation with respect to age, and whose lumbar lordotic Cobb's angles were found to be conserved (high) in old age groups or lost (low) in young age groups.

## 5. Conclusion

In conclusion, our study revealed that a crucial association exists between disc herniation level, age, and angle of lumbar lordosis. Disc herniation was found to occur at higher levels of the spine as age increased and lumbar lordotic Cobb's angle decreased. This finding might be due to flattening of the spine, which begins in the fourth decade at lower lumbar levels and continues till the sixth decade at higher levels. It can also be correlated to trunk muscles imbalance, and the incidence of facet angle asymmetry, which, along with an increase in intervertebral discs stress distribution, would move the lumbar pressure points cranially with load bearing and aging. This would result in disc degeneration and herniation that start initially at the lower lumbar levels, except in cases where lumbar lordosis was found to be outside the predicted measure as implied by age, subsequently leading to unexpected levels of disc herniation.

## Figures and Tables

**Table 1:** Lumbar lordosis and age.

	Total		Group 1		Group 2	
Age	*N*	LLA	*N*	LLA^∗†^	*N*	LLA
30–39	223	32.35°	5	28.6°	218	35.76°
40–49	308	29.8°	11	25.4°	297	30.97°
>70	218	25.26°	115	33.2°	103	24.19°

*Lordotic lumbar angle.

**^†^**The mean values for the lumbar lordotic Cobb's angle for groups 1 and 2 are compared. The third- and fourth-decade groups of patients in group 1 (5 and 11, resp.), who had evidence of a high level (L1-2, L2-3) disc herniation as measured by Cobb's method, unexpectedly show lower mean angles (28.6° and 25.4°, resp.) than the other groups. As for the older patients (>70 years of age), group 1 (115 patients), who had evidence of a low level (L4-5, L5-S1) disc herniation as measured by Cobb's method, shows an unexpectedly higher mean angle (33.2°).
